# Long-wavelength native-SAD phasing: opportunities and challenges

**DOI:** 10.1107/S2052252519002756

**Published:** 2019-04-01

**Authors:** Shibom Basu, Vincent Olieric, Filip Leonarski, Naohiro Matsugaki, Yoshiaki Kawano, Tomizaki Takashi, Chia-Ying Huang, Yusuke Yamada, Laura Vera, Natacha Olieric, Jerome Basquin, Justyna A. Wojdyla, Oliver Bunk, Kay Diederichs, Masaki Yamamoto, Meitian Wang

**Affiliations:** a Swiss Light Source, Paul Scherrer Institut, Villigen PSI, 5232, Switzerland; b Structural Biology Research Center, Photon Factory, Institute of Materials Structure Science, High Energy Accelerator Research Organization, Tsukuba, 305-0801, Japan; c Advanced Photon Technology Division, RIKEN SPring-8 Center, Hyogo 679-5148, Japan; d Laboratory of Biomolecular Research, Department of Biology and Chemistry, Paul Scherrer Institut, Villigen, PSI 5232, Switzerland; e Department of Biochemistry, Max Planck Institute of Biochemistry, Munich, Germany; f Department of Biology, University of Konstanz, Konstanz, 78457, Germany

**Keywords:** single-wavelength anomalous dispersion, native-SAD phasing, Se/S-SAD, UV-laser cutting, crystal shaping, spherical crystals, absorption correction, anomalous scattering factor, structure determination

## Abstract

Native single-wavelength anomalous dispersion (SAD) is an attractive experimental phasing technique as it exploits weak anomalous signals from intrinsic light scatterers (*Z* < 20). The anomalous signal of sulfur in particular, is enhanced at long wavelengths, however the absorption of diffracted X-rays owing to the crystal, the sample support and air affects the recorded intensities. Thereby, the optimal measurable anomalous signals primarily depend on the counterplay of the absorption and the anomalous scattering factor at a given X-ray wavelength. Here, the benefit of using a wavelength of 2.7 over 1.9 Å is demonstrated for native-SAD phasing on a 266 kDa multiprotein-ligand tubulin complex (T_2_R-TTL) and is applied in the structure determination of an 86 kDa helicase Sen1 protein at beamline BL-1A of the KEK Photon Factory, Japan. Furthermore, X-ray absorption at long wavelengths was controlled by shaping a lysozyme crystal into spheres of defined thicknesses using a deep-UV laser, and a systematic comparison between wavelengths of 2.7 and 3.3 Å is reported for native SAD. The potential of laser-shaping technology and other challenges for an optimized native-SAD experiment at wavelengths >3 Å are discussed.

## Abstract 2

Advantages of using a wavelength of 2.7 Å over a wavelength of 1.9 Å for native single-wavelength anomalous dispersion phasing are presented, and potentials of using a wavelength of 3.3 Å are discussed.

## 1. Introduction   

Most of our knowledge about the 3D atomic structure of biological macromolecules is derived directly or indirectly from experimental phasing (EP) in macromolecular crystallography (MX) thanks to the large success of seleno­methio­nine incorporation and heavy-atom derivatization (Hendrickson, 2014 ▸). The emerging native single-wavelength anomalous dispersion (SAD) phasing method has distinct advantages over traditional EP using heavy atoms, but its practical use has so far been limited (Rose *et al.*, 2015 ▸). Indeed, the phasing signal from naturally present light elements in biological macromolecules (*e.g.* sulfur and phospho­rus) is weak in the conventional energy range used at most MX beamlines *i.e.* between 6 and 20 keV (at wavelengths 2.07–0.62 Å) (Djinovic Carugo *et al.*, 2005 ▸). Most attempts at solving native-SAD structures in the early days, including the first native-SAD crambin structure in 1981 (Hendrickson & Teeter, 1981 ▸), were performed using a Cu source at a wavelength of 1.54 Å. Later, a dedicated laboratory X-ray system (Read *et al.*, 2001 ▸; Chen *et al.*, 2004 ▸; Gentry *et al.*, 2005 ▸; Deng *et al.*, 2005 ▸; Kitamura *et al.*, 2008 ▸; Alag *et al.*, 2009 ▸) producing a wavelength of 2.29 Å with a chromium anode was used to solve ∼31 *de novo* structures (Rose *et al.*, 2015 ▸). However, the relatively low flux and large beam size of such sources have limited their application to large and well diffracting crystals. In 2000, the first *de novo* native-SAD structure (obelin; Liu *et al.*, 2000 ▸) was solved with synchrotron radiation using a wavelength of 1.77 Å. Since then, about 60 *de novo* structures have been determined using wavelengths ranging from 1.70 to 2.07 Å (7.3–6.0 keV) at synchrotron beamlines with standard MX sample environments (Weinert *et al.*, 2015 ▸). Recent advances in data-collection methods and multi-crystal averaging have significantly improved the success rate of native SAD using 6 keV X-rays by enabling accurate diffraction-intensity measurement and effective data merging (Liu *et al.*, 2012 ▸; Weinert *et al.*, 2015 ▸; Liu & Hendrickson, 2015 ▸, 2017 ▸; Rose *et al.*, 2015 ▸; Olieric *et al.*, 2016 ▸). However, most systems with low sulfur content and/or low diffraction resolution worse than 3 Å are still out of reach.

**Figure 1:**
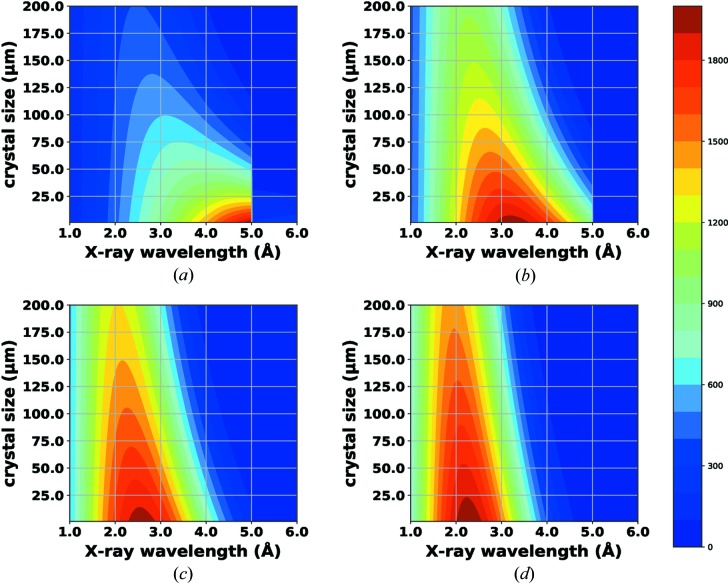
2D contour plots of theoretical anomalous diffraction efficiency for S atoms (shown as a heat map) as a function of X-ray wavelength (*x* axis) and crystal thickness (*y* axis). (*a*) In an ideal experimental condition with ‘naked’ crystals. (*b*) The absorption of 50 µm of solvent around the crystal is included. (*c*) The absorption of 100 mm of air in the scattering path between the crystal and the detector surface is included. (*d*) Both the 50 µm solvent layer and the 100 mm of air are included.

For such challenging cases, it should be advantageous to use energy below 6 keV because the anomalous signal of S and P increases gradually towards lower energy (∝ λ^2^). However, the X-ray absorption by the sample increases as well (cross-section ∝ λ^3^). The counterplay of these two factors produces crystal-size-dependent behavior when searching for the optimal energy, which maximizes the anomalous signal for native SAD (Fig. 1 ▸ and Appendix *A* [App appa]) (Mueller-Dieckmann *et al.*, 2005 ▸; Liu *et al.*, 2013 ▸; Wagner *et al.*, 2016 ▸; Liebschner *et al.*, 2016 ▸). A wavelength of 3 Å appears to be optimal for a 100 µm sized ‘naked’ crystal, *i.e.* one without any surrounding solvent or a loop, in an ideal experiment [Fig. 1 ▸(*a*)]. However, in addition to the crystal itself, any material in the X-ray beampath contributes to absorption and background scattering. Assuming 50 µm of solvent around the crystal and 100 mm of air between the crystal and the detector [Figs. 1 ▸(*b*) and 1 ▸(*c*), and S1 in the Supporting information], the optimum for native SAD shifts towards ∼2 Å [Fig. 1 ▸(*d*)], a wavelength that has been used successfully at standard MX beamlines (Mueller-Dieckmann *et al.*, 2007 ▸; Liu *et al.*, 2012 ▸; Weinert *et al.*, 2015 ▸). Recent attempts towards using longer wavelengths were made at beamline BL-1A at the Photon Factory (PF), Japan and beamline I23 at the Diamond Light Source (DLS), UK, with helium and a vacuum environment, respectively. At beamline BL-1A, the advantage of a wavelength of 2.7 Å over a wavelength of 1.9 Å was demonstrated for both ferredoxin reductase and lysozyme crystals of 100 µm or smaller, while the advantage of using wavelengths of 3.0 Å (4.13 keV energy) and 3.3 Å (3.75 keV energy) remained elusive (Liebschner *et al.*, 2016 ▸). Very recently, a proof-of-principle native-SAD experiment using a wavelength of 4.96 Å (*i.e.* just above the sulfur *K* edge) with a thaumatin crystal was demonstrated for the first time (Aurelius *et al.*, 2017 ▸). In addition to model systems, only a few new structures have been determined using wavelengths ranging from 2.5 to 3.1 Å: death receptor 6 or DR6 (2.7 Å; Ru *et al.*, 2012 ▸) and Lili-Mip (2.7 Å; Banerjee *et al.*, 2016 ▸) at BL-1A in PF; PETase (2.5 Å; Austin *et al.*, 2018 ▸), SSeK3 (Se/S-SAD at 2.77 Å; Esposito *et al.*, 2018 ▸) and ThcOX (3.1 Å; Bent *et al.*, 2016 ▸) at I23 in DLS; and Cdc23^Nterm^ (2.7 Å; Cianci *et al.*, 2016 ▸) at P13 at PETRAIII. These results provide a glimpse into how X-rays with wavelengths longer than 2 Å can be exploited for native-SAD phasing. Indeed, the prospect of native SAD at wavelengths closer to the sulfur *K* edge is very appealing but the technical challenges caused by the increased absorption and scattering, as well as detector efficiency, could be impediments to its wide adoption at synchrotron beamlines. Therefore, we set out a systematic study to identify factors that currently limit the optimal use of longer wavelengths for native SAD and to propose approaches to overcome such limitations.

Both sample thickness – the crystal itself, as well as the loop and surrounding solvent – and absorption correction have to be considered at long wavelengths. While crystal size and morphology are difficult to control precisely during crystallization, crystal ablation using UV-laser-shaping technology (Murakami *et al.*, 2004 ▸) may be used to reduce the sample thickness and to remove extra materials. A deep-UV laser – which can cut polymers such as proteins and fibers and can break chemical bonds by photochemical reactions – was developed for such applications at RIKEN, SPring-8, Japan. It was used to trim crystals from both lysozyme and the membrane protein AcrB mounted on nylon loops under a cryogenic temperature of 100 K (Kitano *et al.*, 2005 ▸). The crystal integrity was shown to be preserved after laser irradiation (Kitano *et al.*, 2005 ▸), which spreads damage only within ∼3 µm of the beam footprint (*Materials and methods* [Sec sec2]). These results clearly show that this technique can shape fragile protein crystals in a more controlled way than mechanical actions such as manual cutting or sonication (de la Cruz *et al.*, 2017 ▸), and is effective in producing various geometric shapes including spheres.

In addition to sample absorption, other factors such as detector performance at low energy, and inaccuracy in data reduction and correction, could result in reduced data quality and compromise the gain in anomalous signal for native SAD at long wavelengths. In this study, the absorption effect for wavelengths >2 Å in native-SAD phasing experiments was assessed systematically. We used a challenging 266 kDa tubulin complex to show the advantages of a 2.7 Å wavelength over a 1.9 Å wavelength for native SAD. We then successfully applied a 2.7 Å wavelength to solve a 86 kDa helicase Sen1 protein using a multi-orientation data-collection protocol (Weinert *et al.*, 2015 ▸). Finally, we exploited the potential of a wavelength of 3.3 Å using spherical lysozyme crystals that have been shaped by the laser (Kitano *et al.*, 2005 ▸). The conditions required to perform an optimal native-SAD experiment at X-ray wavelengths >2 Å, in particular with regard to both sample absorption and detector technology, are discussed.

## 2. Materials and methods   

### 2.1. Sample preparation and crystallization   

#### 2.1.1. Lysozyme   

Lysozyme crystals were produced by the vapor-diffusion method. The protein concentration was 50 m*M* at 50 mg ml^−1^. The lysozyme was solubilised at 50 mg ml^−1^ in 50 m*M* sodium acetate at pH 4.5. The crystals were obtained by mixing 1 µl of the protein with 1 µl of reservoir solution, consisting of 50 m*M* sodium acetate at pH 4.5, 5% PEG MME 5000 and 25% ethyl­ene glycol. Lysozyme crystals of average size 800 × 500 × 400 µm in space group *P*4_3_2_1_2 grew within two days. They were harvested in MiTeGen MicroLoops E and snap-cooled in liquid nitro­gen prior to the laser-shaping experiment.

#### 2.1.2. T_2_R-TTL   

Tubulin-TTL is a multi-ligand globular protein complex (PDB code 4wbn; Weinert *et al.*, 2015 ▸) of size 266 kDa, containing 118 S, 13 P, 2 Cl^−^ and 3 Ca^2+^, which crystallizes in space group *P*2_1_2_1_2_1_. The protein was expressed, purified and crystallized as described elsewhere (Prota *et al.*, 2013 ▸). The needle-like crystals were harvested in Molecular Dimensions ActiLoops with minimum surrounding solvent (<10 µm) and snap-cooled in liquid nitro­gen.

#### 2.1.3. Sen1   

Sen1 is a superfamily 1B (SF1B) helicase protein of 85.7 kDa size (PDB code 5mzn), containing 32 S. Sen1 was expressed and purified from *Escherichia coli* as described elsewhere (Leonaitė *et al.*, 2017 ▸). The Sen1 protein was concentrated to 3 mg ml^−1^ and crystallized at 4°C using the vapor-diffusion method by mixing an equal volume of protein with reservoir solution. The crystallization solution consists of 6% PEG 8000, 8% ethyl­ene glycol and 0.1 *M* HEPES at pH 7.5 buffer. The crystals, in space group *P*2_1_2_1_2, were harvested in Molecular Dimensions ActiLoops with minimum surrounding solvent (10–20 µm) and snap-cooled in liquid nitro­gen.

### 2.2. BL-1A experimental setup   

The long-wavelength native-SAD experiments were carried out at beamline BL-1A at the Photon Factory, KEK, Japan, at X-ray wavelengths of 1.9, 2.7 and 3.3 Å using one or two EIGER 4M detectors enclosed in a helium chamber to overcome the X-ray absorption caused by air. When two EIGER 4M detectors were used, they were configured with V-shape geometry at an adjacent tilt angle of 25°. The detector threshold energy was set to half of the X-ray energy for the 1.9 and 2.7 Å experiments, and 2.3 keV for the 3.3 Å experiment, which corresponds to a threshold of 50, 50 and ∼61%, respectively. BL-1A is equipped with a mini-kappa goniometer with an arm offset of 20°. We used a 40 × 40 µm beam size for all experiments. The flux values are approximately 1.5 × 10^11^, 1.2 × 10^11^ and 1.1 × 10^11^ photons s^−1^ at 1.9, 2.7 and 3.3 Å, respectively.

### 2.3. Native-SAD data collection on T_2_R-TTL and Sen1 crystals at BL-1A   

#### 2.3.1. T_2_R-TTL   

**Figure 2:**
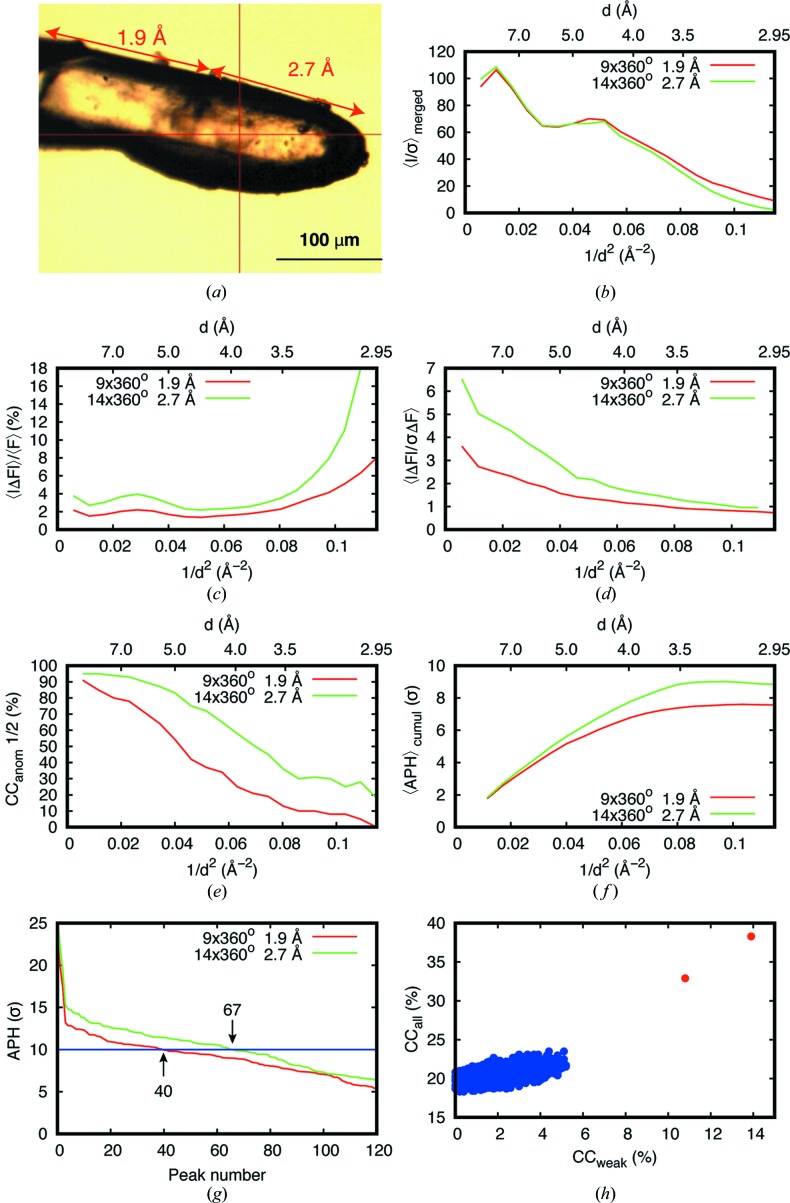
Measurement and comparison of T_2_R-TTL crystal collected at 1.9 and 2.7 Å. (*a*) The crystal mounted on an elliptical Actiloop and the presence of minimum solvents around the crystal. The data-collection positions for each wavelength are marked with red lines with arrows. (*b*) *I*/σ(*I*) values plotted against resolution for datasets collected at both wavelengths. (*c*)–(*f*) 〈|Δ*F*|〉/〈*F*〉, 〈|Δ*F*|/σ(Δ*F*)〉, CC_anom_(1/2) and average anomalous peak height (〈APH〉) values are plotted against the diffraction resolution. (*g*) The anomalous peak heights (APH) are plotted for both wavelengths with dose-equivalent datasets. (*h*) CC_all_ versus CC_weak_ plot from the *SHELXD* solution for the 14 × 360° datasets at 2.7 Å.

We collected native-SAD data from a T_2_R-TTL crystal of size 500 × 70 × 50 µm [Fig. 2 ▸(*a*) and Table 1 ▸] at wavelengths of 1.9 and 2.7 Å. We collected 14 × 360° datasets at different κ angles (Table 1 ▸) at 2.7 Å with 3.4% beam transmission and 21 × 360° datasets at 1.9 Å with 16.5% beam transmission on an EIGER 4M detector placed 60 mm away from the crystal. During data collection, we travelled the longest dimension of the crystal to reduce damage and minimize the systematic errors by introducing fresh crystalline material. We collected all native-SAD datasets from one T_2_R-TTL crystal; the right-hand part of the crystal was collected at 2.7 Å and the left-hand part at 1.9 Å [Fig. 2 ▸(*a*)]. The total doses were estimated as 3.9 MGy and 9.1 MGy for 14 of the 2.7 Å datasets and 21 of the 1.9 Å datasets, respectively.

#### 2.3.2. Sen1   

**Figure 3:**
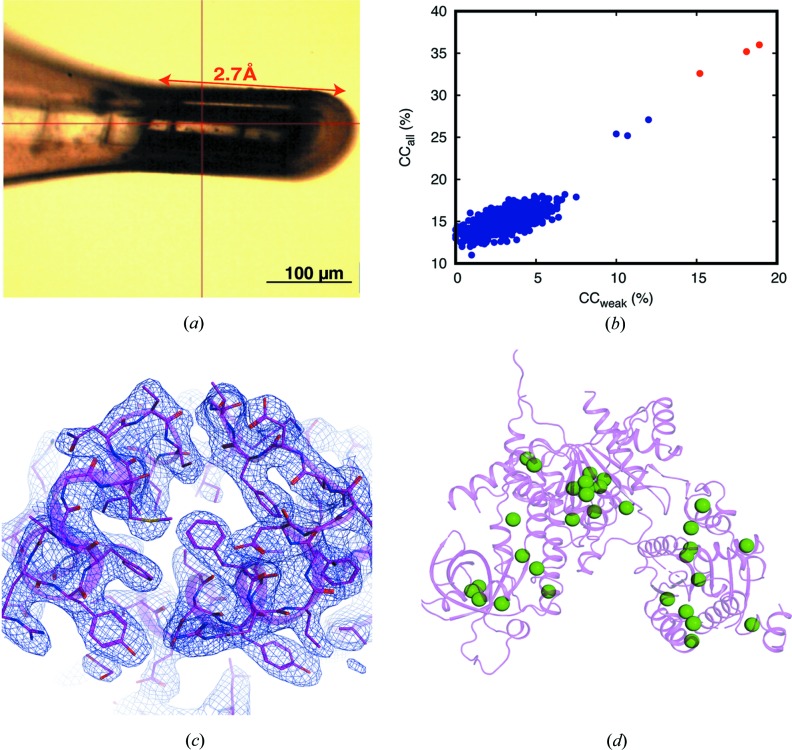
Measurement and native-SAD phasing for Sen1 protein using 2.7 Å. (*a*) The crystal mounted on an elliptical ActiLoop with the data-collection region marked with a red double-headed arrow. (*b*) The CC_all_ versus CC_weak_ plot shows the successful substructure determination by *SHELXD*. (*c*) Experimental phasing map of a selected region of Sen1 after density modification (shown in blue), contoured at 1.0σ along with the C^α^ trace, produced by *CRANK2* (shown as a light-pink colored cartoon representation). (*d*) A cartoon representation of Sen1 protein, with anomalous scatterers (*i.e.* S atoms) highlighted as green spheres.

Native-SAD datasets from the helicase protein Sen1 crystal of size 200 × 100 × 50 µm [Fig. 3 ▸(*a*)] were collected at a wavelength of 2.7 Å on an EIGER 4M detector placed 60 mm away from the crystal. We collected 4 × 360° datasets at different orientations using the mini-kappa goniometer (Table 1 ▸). A beam size of 40 × 40 µm and a beam transmission of 3.4% were used. The total accumulated dose was 1.8 MGy.

### 2.4. Laser-shaping machine and shaping of lysozyme crystals   

**Figure 4:**
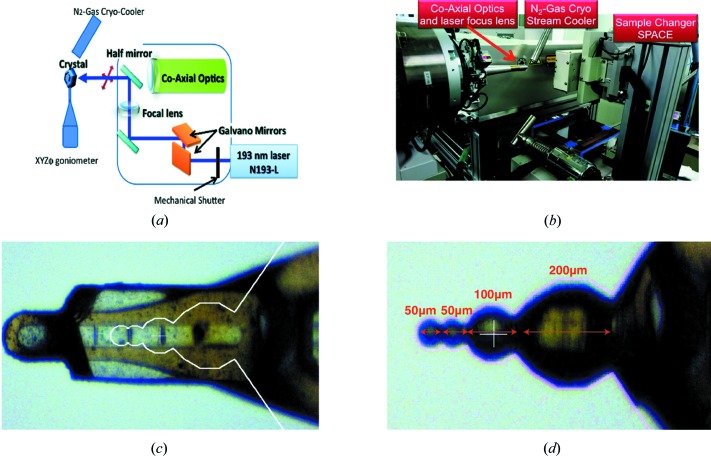
Deep-UV-laser machine setup and laser shaping of lysozyme crystal into spheres of different diameters. (*a*) Schematic diagram of the deep-UV-laser machine along with focusing optics, goniometer and cryojet. (*b*) Top-view of the real-life setup of the deep-UV-laser system at SPring-8, Japan. (*c*) Original large lysozyme crystal of 800 × 500 × 400 µm (before laser cutting) and the white contour was a template made for a spherical shape with precise diameters of each of the spheres, including the error margin for the laser-cutting process. (*d*) The same crystal after laser cutting with a deep-UV laser of wavelength 193 nm. The size of each sphere is written in red and the diameters are marked with corresponding red lines. There are four spheres – two of 50 µm, one of 100 µm and one of 200 µm diameter. The part of the crystal on the extreme right is the ‘unshaped’ region, along with the base of the original loop. A supplementary movie of the laser-cutting process is also available in the Supporting information.

A compact, fast and user-friendly laser shaping system (Murakami *et al.*, 2004 ▸; Kitano *et al.*, 2004 ▸, 2005 ▸) was developed at RIKEN, SPring-8, Japan, to trim crystals in to various shapes [Fig. 4 ▸(*a*)]. The deep-UV laser, which uses an NSL-193L laser source (Nikon) of wavelength 193 nm and a pulse duration of ∼1 ns (Kitano *et al.*, 2005 ▸), operates at an energy of 8.0 µJ. High-speed scanning galvanometer mirrors focus the beam to 4.6 × 3.9 µm (*H* × *V*, FWHM) [Fig. S2(*b*)]. Crystals were mounted on a high-precision single-axis goniometer with *X*/*Y*/*Z* linear stages using the SPACE sample changer (Murakami *et al.*, 2012 ▸) and kept at 100 K under a cryostream [Fig. 4 ▸(*b*)]. We shaped one lysozyme crystal (800 × 500 × 400 µm) mounted on a MicroLoops ETM (MiTeGen) into four connected spheres with diameters of 50, 50, 100 and 200 µm [Figs. 4 ▸(*c*) and 4 ▸(*d*), and Supplementary movie S1] and another lysozyme crystal into a cylindrical shape of 500 × 50 × 50 µm. The procedure took about 20 min per crystal.

Irradiation damage of the deep-UV laser was evaluated with a micro-focused X-ray beam at beamline BL32XU at SPring-8, Japan (Hirata *et al.*, 2013 ▸) using a cytochrome *c* oxidase crystal (Tsukihara *et al.*, 1995 ▸). The crystal was shaped using the deep-UV system with lines [Figs. S2(*a*) and S2(*b*)] and then rastered using a beam of 1.0 × 5.0 µm (*H* × *V*, FWHM) at a wavelength of 1.0 Å and a flux of 6.0 × 10^9^ photons s^−1^. The diffraction images were processed using *SHIKA* (Hirata *et al.*, 2014 ▸). We observed a loss of diffraction over an ∼10 µm thick area [Fig. S2(*c*)], which implies that the radiation damage extends by ∼3 µm on each side of the deep-UV laser-beam footprint.

### 2.5. Dose-normalization measurement   

Dose-normalization analysis was carried out using a cylindrical lysozyme crystal that had been shaped by the laser [Fig. S3(*a*)] of size 550 × 50 × 50 µm, to compare flux and beam transmission between the 2.7 and 3.3 Å wavelengths. The X-ray dose was estimated based on intensity decays as measured by the relative *B* factors, followed by linear curve fitting. At 2.7 Å, 8 × 360° datasets were collected with 12.4% beam transmission per crystal position. Each dataset (1 × 360°) was collected with 0.2° and 0.1 s per step, corresponding to an accumulated dose of 3.87 MGy [Fig. S3(*b*)]. We repeated the same experiment at 3.3 Å with 2.27% beam transmission at a different crystal position [Fig. S3(*a*)]. Here, the accumulated dose was 0.97 MGy per 360° dataset [Fig. S3(*c*)]. The dose ratio between the two wavelengths (2.7 versus 3.3 Å) was therefore ∼4. Thus, 12.4/4 = 3.1% beam transmission at 2.7 Å and 2.27% beam transmission at 3.3 Å, which should deposit similar doses on the sample. These beam transmissions were used in the subsequent experiments where anomalous diffraction efficiencies at 2.7 and 3.3 Å were compared.

### 2.6. Data collection on laser-shaped lysozyme crystals   

A lysozyme crystal was mounted on the mini-kappa goniometer at beamline BL-1A, KEK Photon Factory, Japan (Fig. 4 ▸). The shaped crystal consisted of four connected spheres: two 50, one 100 and one 200 µm in diameter. Datasets with comparable dose were collected with the bottom EIGER 4M detector in a V-shape configuration at wavelengths of 2.7 and 3.3 Å with beam transmission of 3.10 and 2.27%, respectively. Two 360° datasets were collected from each of the 50 µm and each of the 100 µm diameter spheres, while only one 360° dataset was collected from the 200 µm diameter sphere at each wavelength. The accumulated doses per dataset at both wavelengths were about 0.9, 0.45 and 0.225 MGy per dataset for 50, 100 and 200 µm spheres, respectively. All the 2.7 Å datasets were collected after the 3.3 Å datasets. All data were collected using oscillation steps of 0.2° and an exposure time of 0.1 s per step.

### 2.7. Data processing, phasing and refinement   

All diffraction data were processed with *XDS* (Kabsch, 2010*a* ▸) and scaled with *XSCALE* (Kabsch, 2010*b* ▸). Anomalous data were analyzed with *SHELXC/D/E* (Sheldrick, 2010 ▸) using the *HKL2MAP* interface (Pape & Schneider, 2004 ▸). The substructure determination was performed using *SHELXD* (Schneider & Sheldrick, 2002 ▸), followed by phasing, density modification and automatic model building using *CRANK2* (Skubák & Pannu, 2013 ▸). The final refinements were carried out using *phenix.refine* (Afonine *et al.*, 2012 ▸). The anomalous peak heights were calculated by *ANODE* (Thorn & Sheldrick, 2011 ▸).

#### 2.7.1. T_2_-TTL   

For T_2_R-TTL, both 1.9 and 2.7 Å datasets were processed with *XDS*. The multiple datasets at each wavelength were scaled with *XSCALE*. To have common reflections for a direct comparison between datasets at two wavelengths, all data were truncated to 2.95 Å resolution, which resulted in nearly the same unique reflections (only 0.4% difference) (Tables S1 and S2). The structure was solved from 14 × 360° datasets collected at 2.7 Å. The substructure was determined using *SHELXD* (Schneider & Sheldrick, 2002 ▸) by searching for 100 sites at a resolution cutoff of 3.5 Å with an *E*_min_ value of 1.3 and 10 000 trials. This yielded a CFOM of 52.3% (CC_all_ = 38.3% and CC_weak_ = 13.9%). The substructure sites were parsed to the *CRANK2* pipeline (Skubák & Pannu, 2013 ▸), which completed the sites and carried out phasing, density modification and automatic model building. The final structure was refined at 2.95 Å resolution in *phenix.refine* (Afonine *et al.*, 2012 ▸) with resulting *R*_work_ of 17.0% and *R*_free_ of 20.8% (Table 1 ▸).

#### 2.7.2. Sen1   

For Sen1, the crystal diffracted to 2.8 Å at a wavelength of 2.7 Å. The structure was determined from 4 × 360° datasets. *SHELXD* successfully produced a substructure of 22 sites at a resolution cutoff of 3.3 Å with 1 000 tries, resulting in a CFOM of 54.9% (CC_all_ = 36.0% and CC_weak_ = 18.9%). *CRANK2* (Skubák & Pannu, 2013 ▸) automatically built 692 out of 720 residues. The final refinement of Sen1 structure was performed at 2.95 Å resolution with *phenix.refine* (Afonine *et al.*, 2012 ▸), resulting in final *R*_work_ of 16.9% and *R*_free_ of 21.3% (Table 1 ▸).

#### 2.7.3. Laser-shaped lyozyme   

For laser-shaped lysozyme, a V-shaped detector configuration allowed diffraction resolutions of 2.3 and 2.8 Å at wavelengths of 2.7 and 3.3 Å, respectively. During data processing in *XDS* (Kabsch, 2010*b* ▸), STRICT_ABSORPTION_CORRECTION was set to TRUE, AIR was set to ZERO and we manually defined a mask to eliminate shadowed regions caused by overlap between two adjacent detectors in a V configuration. Only the data from the bottom detector were used in data analysis. In the study of the effect of sample thickness at each wavelength, data were used to the full resolution (Tables S3 and S4). In the direct comparison between 2.7 and 3.3 Å datasets, only common reflections to 2.8 Å were used. These reflections were selected using a custom script from the unmerged data in *INTEGRATE.HKL* before scaling by the CORRECT routine in *XDS* (Table S5).

## 3. Results   

### 3.1. Dose-normalized intensity across wavelength   

To study the optimal wavelength for native-SAD phasing, the measured anomalous signal per absorbed X-ray dose needs to be compared at different wavelengths. This dose-normalized anomalous efficiency [Appendix *A* [App appa] and equation (4)] can be approximated by a dose-normalized diffracted intensity [Appendix *A* [App appa] and equation (3)] multiplied by the anomalous scattering factor (*f*′′), assuming the X-ray dose is proportional to the absorbed photon energy. Equation (4) suggests that 1.9, 2.7 and 3.3 Å wavelengths are optimal for a crystal size of >200, 125 and 75 µm, respectively, under ideal experimental conditions (*i.e.* no surrounding solvent, loop or air, perfect X-ray beam and detector) [Fig. 1 ▸(*a*)]. The intensity in equation (3) can be used to calculate the theoretical intensity ratio at different wavelengths with an equivalent dose. For example, the expected intensity ratio is 1.16 for a 70 µm thick crystal between 1.9 and 2.7 Å, and is 1.15 for a 50 µm thick crystal between 2.7 and 3.3 Å. These intensity ratios were compared with experimentally observed intensity ratios between two wavelengths – 1.9 versus 2.7 Å (*i.e.* T_2_R-TTL) and 2.7 versus 3.3 Å (*i.e.* laser-shaped lysozyme spheres) – to validate the dose normalization.

To study the crystal size dependence of native SAD at a given wavelength using spherically shaped lysozyme crystals, the theoretical diffracted intensity was estimated by calculating both diffraction volume and absorption correction numerically (Appendix *B* [App appb]). Then the theoretical diffracted-intensity ratios across various thicknesses of the crystal were calculated (Table S6) and compared with experimentally observed intensity ratios across different diameters of the laser-shaped lysozyme spheres in Section 3.3.

### 3.2. Advantage of a wavelength of 2.7 Å over 1.9 Å for 100 µm or smaller crystals   

#### 3.2.1. T_2_-TTL   

We used the tubulin complex T_2_R-TTL (Prota *et al.*, 2013 ▸) to assess the advantages of native SAD at 2.7 Å. A T_2_R-TTL needle-shaped crystal (500 × 70 × 50 µm) was mounted on an elliptical ActiLoop (Molecular Dimensions) with minimum surrounding solvent [Fig. 2 ▸(*a*)]. Using different crystal orientations (Weinert *et al.*, 2015 ▸), 21 × 360° and 14 × 360° datasets were collected on two crystal positions using 1.9 and 2.7 Å, respectively [Fig. 2 ▸(*a*)]. We used the ratio of observed diffraction intensities between the two wavelengths to achieve a dose-normalized comparison. The mean intensity ratio of the two 360° datasets at the two wavelengths was 1.8 (Fig. S4), while the theoretical dose-normalized intensity ratio was estimated to be 1.16 (Appendix *A* [App appa]). Therefore, 9 × 360° datasets at 1.9 Å and 14 × 360° datasets at 2.7 Å had a comparable dose [Fig. 2 ▸(*b*)]. We observed the expected Bijvoet ratio (1.5 and 2.8% at 1.9 and 2.7 Å, respectively) in the measured anomalous differences (〈|Δ*F*|〉/〈*F*〉) [Fig. 2 ▸(*c*)] and an abrupt rise of 〈|Δ*F*|〉/〈*F*〉 above 3.5 Å resolution in the 2.7 Å dataset, which does not represent the true anomalous signal but instead indicates that the anomalous signal is buried in the exceeding errors in the weak data at high resolution (Dauter *et al.*, 2002 ▸). The corresponding increase of 〈|Δ*F*|/σ(Δ*F*)〉 is also in a good agreement with the ∼86% gain in anomalous scattering factor *f*′′ of sulfur at the two wavelengths [Fig. 2 ▸(*d*)]. In addition, the higher anomalous signal in the 2.7 Å dataset is very visible in the half-dataset anomalous correlation [Fig. 2 ▸(*e*)] and the average anomalous peak heights (〈APHs〉) [Fig. 2 ▸(*f*)]. The merged data at 2.7 and 1.9 Å gave 67 and 40 anomalous peaks above 10σ, respectively [Fig. 2 ▸(*g*)]. Here, the 2.7 Å wavelength data produced successful substructures using *SHELXD* [Fig. 2 ▸(*h*)], and 2 152 out of 2 363 residues could be built correctly using density modification and automatic model building in *CRANK2* (Skubák & Pannu, 2013 ▸). The final structure was refined to 2.95 Å resolution with an *R*_work_ and an *R*_free_ of 17.0% and 20.8%, respectively, using *phenix.refine* (Afonine *et al.*, 2012 ▸). Native-SAD phasing was also possible at 1.9 Å but only by merging 21 datasets, which constituted more than double the dose used at 2.7 Å. This example clearly illustrates the benefits of performing native SAD at a wavelength of 2.7 Å over 1.9 Å for crystals with 100 µm diameter or less and with minimum extra surrounding materials in the absence of air absorption.

#### 3.2.2. Sen1   

We applied native-SAD measurement using a wavelength of 2.7 Å on Sen1: an 85.7 kDa helicase protein with 32 S atoms, involved in the termination of non-coding transcription processes. The Sen1 crystal measured as 220 × 100 × 50 µm was carefully mounted on an elliptical loop with minimum surrounding solvent [Fig. 3 ▸(*a*)]. Using 4 × 360° datasets collected at multiple crystal orientations and merged together, the substructure was readily solved by *SHELXD* (Schneider & Sheldrick, 2002 ▸) using a 3.3 Å resolution cutoff [Fig. 3 ▸(*b*)]. The subsequent density improvement and phasing in *CRANK2* pipeline (Skubák & Pannu, 2013 ▸) produced an interpretable map of excellent quality [Figs. 3 ▸(*c*) and S5]. We traced 692 residues successfully and the structure [Fig. 3 ▸(*d*)] was refined to 2.95 Å resolution, resulting in an *R*_work_/*R*_free_ of 16.9%/21.3% using *phenix.refine* (Afonine *et al.*, 2012 ▸).

### 3.3. Native SAD with spherical laser-shaped crystals at 2.7 and 3.3 Å   

Upon establishing the benefits of using 2.7 Å for native SAD, we next explored the potential of an even longer wavelength of 3.3 Å. Theoretically (Appendix *A* [App appa] and Fig. 1 ▸), the sample absorption can be detrimental in abstracting accurate anomalous signals at such a wavelength. We therefore carried out a systematic study to compare the quality of datasets collected at both 2.7 and 3.3 Å using a lysozyme crystal with various thicknesses. Using a deep-UV laser (*Materials and methods* [Sec sec2.4]), we shaped a large lysozyme crystal into connected spheres of 50, 100 and 200 µm diameter (Fig. 4 ▸ and Supplementary movie S1). An added benefit of a spherical shaped crystal is that it minimizes the angular dependence of absorption (Appendix *B* [App appb]). Indeed, the data-processing statistics with and without absorption correction are very similar for the spherical lysozyme crystals except for the 200 µm sphere, where the data quality improved slightly with absorption correction (Fig. S6).

**Figure 5:**
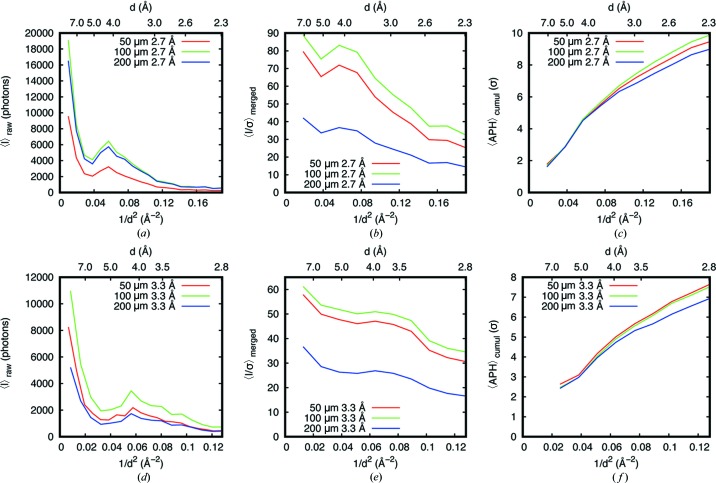
Comparison of data statistics among different spheres of different diameters at wavelengths of 2.7 and 3.3 Å. (*a*) and (*b*) Observed diffracted intensities and *I*/σ(*I*) over resolution shells at 2.7 Å. (*c*) Cumulative average anomalous peak height 〈APH〉 as a function of resolution at 2.7 Å. (*d*) and (*e*) Observed diffracted intensities and *I*/σ(*I*) over resolution shells at 3.3 Å. (*f*) Cumulative 〈APH〉 as a function of resolution at 3.3 Å.

To understand the sample absorption effect, we first compared datasets from 50, 100 and 200 µm spheres at 2.7 Å. Diffraction intensities are plotted in Fig. 5 ▸(*a*). Their ratios were about 2.0 (100:50 µm) and 1.9 (200:50 µm), in good agreement with the theoretical values of 1.9 and 2.1 (Table S6). However, 〈*I*/σ(*I*)〉 values showed very different behavior [Fig. 5 ▸(*b*)], *e.g.* ratios of 〈*I*/σ(*I*)〉 between 100 and 50 µm datasets were less than the expected 1.41 (square root of 2) from Poisson statistics, particularly at low to medium resolutions. The 200 µm dataset had much lower 〈*I*/σ(*I*)〉 compared with the other two datasets. A similar trend is also observed in the 〈APHs〉 [Fig. 5 ▸(*c*)]. We conclude that the sample absorption for crystals of 100 µm or larger (44% and 70% for 100 and 200 µm crystals, respectively) and the inaccuracy in their corrections in data processing inflate the σ(*I*) estimation, which further diminishes the benefits of the increased diffraction volume. Therefore, in order to profit from the improved *f*′′ at a wavelength of 2.7 Å for native SAD, crystal size of 100 µm or smaller should be used, as demonstrated here with T_2_R-TTL (70–50 µm diameter) and Sen1 (50–100 µm diameter).

Similar analyses were carried out for 3.3 Å data. The intensity ratios (100:50 µm and 200:50 µm) again followed the theoretical values (observed 1.5 and 0.7 versus theoretical 1.4 and 0.9, respectively) [Fig. 5 ▸(*d*)]. We suspected that the small differences between theoretical and experimentally measured values were caused by a slight miscentering of the X-ray beam with respect to the crystal. As expected, the gain in diffraction volume is further reduced by the excessive absorption at this wavelength (65 and 89% for 100 and 200 µm crystals, respectively). Therefore, the 〈*I*/σ(*I*)〉 values only increased slightly between the 50 µm dataset and the 100 µm dataset and both datasets have comparable APHs [Figs. 5 ▸(*e*) and 5 ▸(*f*)]. The 〈*I*〉, the 〈*I*/σ(*I*)〉 and the anomalous peak height were lowest for the 200 µm dataset. As clearly shown here, small crystals (<50 µm) are a prerequisite to take full benefit of native-SAD phasing at 3.3 Å and longer wavelength.

**Figure 6:**
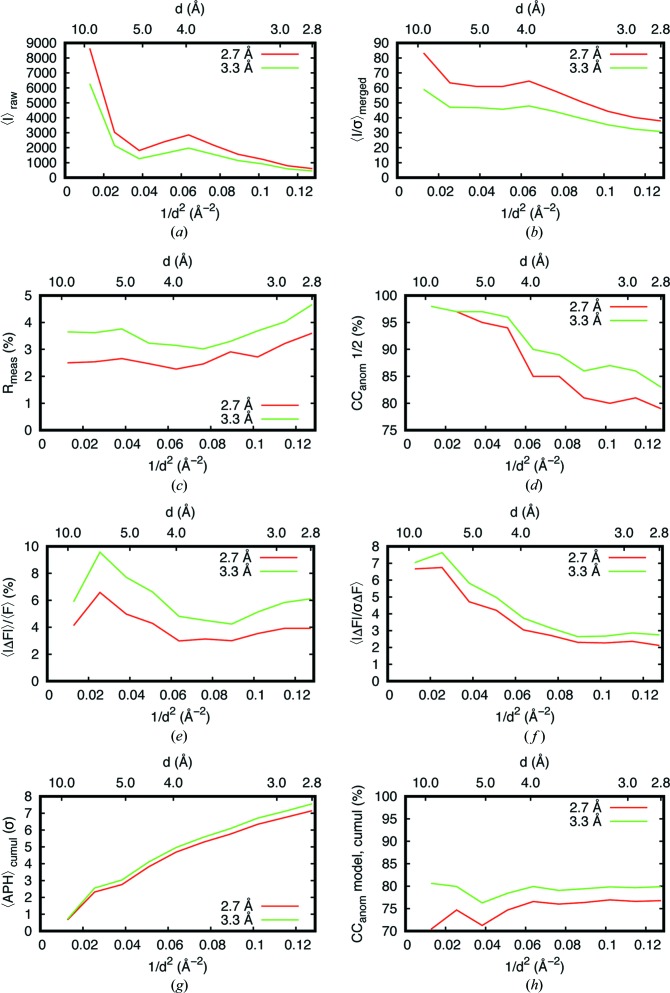
Comparison of data statistics between wavelengths of 2.7 and 3.3 Å on a 50 µm diameter lysozyme sphere. (*a*)–(*f*) Observed diffracted intensities (〈*I*〉), *I*/σ(*I*), *R*_meas_, CC_anom_(1/2), 〈|Δ*F*|〉/〈*F*〉 and 〈|Δ*F*|/σ(Δ*F*)〉 over resolution shells. (*g*) and (*h*) Cumulative average anomalous peak height 〈APH〉 and correlation coefficient between the observed anomalous difference and the calculated anomalous difference from a refined model as a function of resolution.

We then attempted a comparison between 2.7 and 3.3 Å using two datasets from the same 50 µm lysozyme sphere collected with a comparable accumulated X-ray dose (see Section 2.5 for experimental dose measurement). The 2.7 Å dataset had higher observed intensities by about 40% compared with the 3.3 Å dataset [Fig. 6 ▸(*a*)], which is much higher than the theoretical dose-normalized intensity ratio of 1.15 (Appendix *A* [App appa]). The corresponding 〈*I*/σ(*I*)〉 was also much higher at 2.7 Å [Fig. 6 ▸(*b*)]. We attributed these differences to the lower detector efficiency at 3.3 Å, caused by both energy threshold (61%) and absorption from the non-sensitive surface layers. Indeed, the thin aluminium and silicon layers on the sensor, together with a Mylar window in front of the detector absorb as much as 20–30% more photons at 3.3 Å than at 2.7 Å (Donath *et al.*, 2013 ▸). In terms of accuracy, the *R*_meas_ values of the 3.3 Å dataset were slightly higher than that of the 2.7 Å dataset [Fig. 6 ▸(*c*)]. This is likely caused by the so-called ‘corner effect’ from hybrid pixel-array photon-counting detectors (HPCs), which could inflate the *R*_meas_ by introducing systematic intensity-measurement errors, particularly when the detector energy threshold is above 50% as was the case at 3.3 Å (Leonarski *et al.*, 2018 ▸). In addition, the inaccuracy of absorption correction in data processing could also reduce the accuracy of long-wavelength data.

Nevertheless, the 3.3 Å dataset featured higher anomalous signal as measured by the half-dataset anomalous correlation [Fig. 6 ▸(*d*)]. The observed 〈|Δ*F*|〉/〈*F*〉 values were as expected from the Bijvoet ratio estimations (∼3.5 and ∼5% at 2.7 and 3.3 Å, respectively) [Fig. 6 ▸(*e*)]. The higher anomalous difference at low resolution (∼10–5 Å) was caused by the enhanced contribution from the four di­sulfides in lysozyme, unresolved at that resolution. The increase in 〈|Δ*F*|〉/〈*F*〉 at 3.3 Å compared with 2.7 Å was in accordance with the 40% gain in the anomalous scattering factor *f*′′ of sulfur [Fig. 6 ▸(*e*)]. The corresponding 〈|Δ*F*|/σ(Δ*F*)〉 also increased at 3.3 Å but to a lower extent [Fig. 6 ▸(*f*)], indicating the higher noise in the 3.3 Å data as explained earlier. Overall, the 〈APH〉 was improved by 0.5σ to 2.8 Å resolution [Fig. 6 ▸(*g*)] and correlations between the observed anomalous differences (Δ*F*_obs_) and the calculated one (Δ*F*_calc_) from the refined model were improved by a few percent [Fig. 6 ▸(*h*)]. We also noticed that the APH for di­sulfide was clearly lower at 2.7Å than at 3.3 Å (7.1σ versus 8.1σ) while the APH was higher for Met at 2.7 Å (8.7σ versus 8.1σ) and comparable for Cl (6.7σ) at both wavelengths. With a total accumulated dose of ∼2–3 MGy, we attribute this difference to radiation damage on the sensitive disulfide bridges (Murray & Garman, 2002 ▸) because the 2.7 Å dataset was collected after the 3.3 Å dataset formed the same 50 µm crystal. Therefore, when taking the radiation-damage effect into consideration, the obtained anomalous signal improvement at 3.3 Å was found to be marginal in this particular experiment. Further improvement in absorption correction and detector performance at such wavelength is needed in order to harness the gain in *f*′′ for native-SAD phasing fully.

## 4. Discussion   

Optimization of native-SAD phasing experiments at wavelengths >2 Å is being addressed at dedicated MX beamlines with reduced air absorption and scattering effects, as well as special detector geometry. However, so far there has been little research into the adverse effect of sample absorption, as well as detector efficiency at such long wavelengths.

In this work, performed at beamline BL-1A at the Photon Factory with two real-life targets T_2_R-TTL and Sen1, we have demonstrated that the increased anomalous signal at 2.7 Å (sulfur *f*′′ = 1.5 e^−^) over shorter wavelengths can be harnessed effectively as long as the crystal dimension in the beam path is smaller than 100 µm, and extra material around the crystal and air scattering are minimized. Both Sen1 and T_2_R-TTL can diffract to about 2.4 Å resolution, but only data up to 3 Å were collected and successfully used for phasing. Therefore, native SAD at a wavelength of 2.7 Å has the potential to reach targets with lower S-atom content and/or lower diffraction resolution where enhanced anomalous signals are needed. While the specialized sample environment at BL-1A is essential, the results reported here also clearly highlight that both the crystal thickness and surrounding materials – loop and solvent – should to be carefully considered prior to data collection at long wavelengths.

Despite an ∼40% increase in anomalous signal compared with 2.7 Å (*f*′′ = 2.1 e^−^ versus 1.5 e^−^), the potential of native SAD at wavelengths of 3.3 Å and beyond is limited in practice to crystals of 50 µm or smaller in size. We have shown in this study that spherical laser-shaping offers an appealing solution to tackle absorption effects by both realizing a fine control of the sample thickness and simplifying absorption-correction procedures. Indeed, the absorption effect is then identical for reflections at a given scattering angle when a spherically homogeneous crystal is illuminated by an X-ray beam with a symmetric profile at the center of the sphere. Here, an added benefit is that the angular dependency of the absorption can be numerically calculated and applied (Appendix *B* [App appb]). While X-ray tomography has been attempted to reconstruct the shape and volume of macromolecular crystals (Brockhauser *et al.*, 2008 ▸), including the loop and surrounding materials, the correction of the absorption has remained empirical (Blessing, 1995 ▸) in most data-processing software suites and relies largely on the collection of data with high multiplicity and in multiple crystal orientations (Liu *et al.*, 2012 ▸; Weinert *et al.*, 2015 ▸). The possibility to use UV-laser ablation to both remove non-diffracting materials and to shape crystals as spheres is therefore an interesting tool to better deal with absorption effects at long wavelength.

While it may be possible to shape large crystals using UV-laser ablation, microcrystals still remain a challenge for long-wavelength native-SAD phasing. Thanks to recent developments in serial crystallography (SX) methods at both X-ray free-electron lasers and synchrotrons, sample delivery of microcrystals benefits from low scattering background solid-supports, which have been designed to facilitate crystal loading with minimum solvent (Meents *et al.*, 2017 ▸; Owen *et al.*, 2017 ▸; Huang *et al.*, 2015 ▸; Warren *et al.*, 2015 ▸; Huang *et al.*, 2016 ▸; Wierman *et al.*, 2013 ▸; Baxter *et al.*, 2016 ▸; Sui *et al.*, 2016 ▸). The subsequent serial data collection and data-merging methods have been adapted as well (Zander *et al.*, 2015 ▸; Hirata *et al.*, 2013 ▸; Wojdyla *et al.*, 2018 ▸; Yamashita *et al.*, 2018 ▸; Huang *et al.*, 2018 ▸; Basu *et al.*, 2019 ▸). These developments are particularly relevant to small membrane-protein crystals, for which *de novo* phasing is in demand. The recent native-SAD phasing of the membrane protein PepT_st_ with an SX approach required data from about 2 000 microcrystals (10–20 µm) collected at 6 keV. In contrast, only about 100 Se-Met PepT_st_ crystals of similar size were needed for Se-SAD (Huang *et al.*, 2018 ▸). Given the *f*′′ values for S at 4 keV compared with Se at 12.67 keV, (1.8 e^−^ and 3.8 e^−^ respectively) we estimate an order of magnitude less PepT_st_ microcrystals to be required for solving PepT_st_ by native-SAD phasing at 4 keV or lower energies.

Another obstacle of native-SAD phasing at long wavelength is the detector inefficiency. Detection of low-energy photons is challenging for HPCs – the current standard in MX beamlines. The performance of the EIGER 4M detector used in this study was indeed affected by the lowest reachable energy threshold, inaccuracy in threshold calibration (Leonarski *et al.*, 2018 ▸) and absorption from an ∼1 µm Al/Si layer on the surface of the silicon sensor, as well as from a protective 20 µm-thick Mylar foil. Unfortunately, the latter effect becomes more pronounced for high-angle reflections because of parallax effects but can be minimized using a curved detector (Wagner *et al.*, 2016 ▸) or flat detectors in a V-shape configuration. Note that the faster intensity decay towards a high diffraction angle caused by this parallax can also induce artifacts in data processing, *e.g.* inflating the Wilson *B* factor. As an alternative to HPC technology, new hybrid charge integrating technology is being developed and is expected to perform better at low energy (Leonarski *et al.*, 2018 ▸).

Overall, this work highlights the 2.7 Å wavelength as a very suitable energy with current instrumentation for sample thicknesses ≤ 100 µm when mounted appropriately. In addition, it emphasises that minimization of X-ray absorption by careful sample preparation or accurate control of sample thickness and shape by laser ablation, together with improved detector technology, will be instrumental in realising the full potential of long wavelengths (>3 Å) for solving challenging novel structures using native-SAD phasing.

## Supplementary Material

Supplementary tables, figures, description of movie and mathematica script. . DOI: 10.1107/S2052252519002756/jt5032sup1.pdf

Supplementary movie. DOI: 10.1107/S2052252519002756/jt5032sup2.mp4

PDB reference: T2R-TTL, 6i5c

PDB reference: Sen1, 6i59

## Figures and Tables

**Table 1:** Data collection and refinement statistics for T_2_R-TTL and Sen1 native-SAD experiments at a wavelength of 2.7 Å Values in parenthesis represent the highest resolution shell.

Protein	T_2_R-TTL	Sen1
PDB entry	6i5c	6i59
Data collection		
Photon energy (keV)	4.6	4.6
Beam size (µm^2^)	40 × 40	40 × 40
Flux (photons^−1^)	4.1 × 10^9^	4.1 × 10^9^
Space group	*P*2_1_2_1_2_1_	*P*2_1_2_1_2
Unit-cell dimensions (Å)	*a* = 104.24, *b* = 156.83, *c* = 179.54	*a* = 69.11, *b* = 91.05, *c* = 172.07
Oscillation angle (°)	0.2	0.2
Exposure time (s)	0.1	0.1
Total range (°)	14 × 360	4 × 360
Detector distance (mm)	60	60
Total dose (MGy)	3.9	1.8
κ angles (°)	0–65; Δ = 5°	0–30; Δ = 10°
No. of crystal positions	1	1
Structure		
Crystal size (µm^3^)	500 × 70 × 50	220 × 100 × 50
Molecular weight (kDa)/No. of residues	266/2363	85.7/720
Monomer/asymmetric unit	1	1
No. of scatterers	118 S, 13 P, 2 Cl, 3 Ca	32 S
Bijvoet ratio (%)	1.53	1.39
Phasing		
Resolution (Å)	50–2.95 (3.03–2.95)	50–2.95 (3.03–2.95)
No. of unique reflections	62640 (4564)	23557 (1653)
No. of total reflections	11135360 (625357)	1157226 (62626)
Multiplicity	177.8 (137.0)	49.1 (37.9)
Completeness (%)	99.9 (99.5)	99.1 (95.2)
*R*_meas_ (%)	15.3 (289.1)	3.18 (42.79)
CC_1/2_ (%)	100 (82.3)	99.9 (69.3)
〈*I*/σ(*I*)〉	52.23 (3.29)	31.08 (1.66)
Mosaicity (°)	0.17	0.17
*SHELXD* resolution cut-off (Å)	3.5	3.3
CC_all_/CC_weak_	38.3/13.9	36.0/18.9
Solvent content (%)	56.3	60.9
Refinement		
*R*_work_/*R*_free_ (%)	17.0/20.8	16.9/21.3
RMS deviations		
Bond lengths (Å)	0.003	0.003
Bond angles (°)	0.692	0.658
Wilson *B* factor (Å^2^)	77.9	88.2
Average *B* factor (Å^2^)		
All atoms	73.5	88.8
Macromolecules	73.7	83.4
Ligands	72.0	130.2
Solvent	66.3	102.8
Clashscore	4.0	6.5
Ramachandran plot		
Favored (%)	98.0	97.2
Allowed (%)	2.0	2.8
Outliers (%)	0	0

## Appendix A Theory of the optimal wavelength for native SAD

There are theoretical foundations to the optimal X-ray wavelengths for native-SAD phasing (Arndt, 1984 ▸; Polikarpov *et al.*, 1997 ▸; Hendrickson, 2013 ▸; Wagner *et al.*, 2016 ▸). Here, we briefly revisit the theory and add considerations on the absorption by both non-crystalline materials around the crystal and the air in the diffraction beam path. Optimal native-SAD data collection primarily relies on two inter-dependent variables – the materials in the X-ray beam path and the X-ray wavelength. The diffraction efficiency (*I*_E_) defined as the integrated intensity per absorbed energy in a crystal bathed in an X-ray beam (Arndt, 1984 ▸; Polikarpov *et al.*, 1997 ▸) is expressed as

where *t*_xtal_ is the crystal dimension, λ is the X-ray wavelength, and μ_xtal_ is the linear absorption coefficient and is estimated to be 0.32 λ^3^ mm^−1^ for a lysozyme crystal. The exp(−μ_xtal_ *t*_xtal_) term is the X-ray transmittance of the path through the crystal. The 1 − exp(−μ_xtal_ *t*_xtal_) term is the absorption, which is approximately related to the X-ray induced radiation damage. For the scenario where the X-ray beam is smaller than the crystal, *I*_E_ becomes

where *t*_beam_ is the X-ray beam dimension. This case is close to experiments described in this study and its detailed treatment for spherical crystals is given in Appendix *B* [App appb].

Next, we include the solvent around the crystal and the air in the X-ray path from the crystal to the detector,

where *t*_sol_ is thickness caused by surrounding solvent/mother liquor around the crystal (we assume that crystal and solvent have similar linear absorption coefficient), μ_air_ is the linear absorption coefficient of air (3.3 × 10^−4^ mm^−1^ Å^−3^), and *t*_air_ is the path length of air between the crystal and the detector. The  term is constant for this experiment and was removed from equation (3).

Based on equation (3), the expected intensity ratio is 1.16 for a given dose between wavelengths of 1.9 and 2.7 Å using an ∼70 µm thick crystal of T_2_R-TTL. Similarly, for a 50 µm diameter spherical lysozyme crystal that had been shaped by the laser, the expected dose-normalized intensity ratio between wavelengths of 2.7 and 3.3 Å is 1.15.

When searching for the optimal wavelength for native-SAD phasing, anomalous diffraction efficiency (Δ*I*) is used as a metric and is defined as the diffraction efficiency multiplied by the anomalous scattering factor *f*′′ (Hendrickson, 2013 ▸; Wagner *et al.*, 2016 ▸; Liebschner *et al.*, 2016 ▸),

Based on equation (4), anomalous diffraction efficiencies were visualized in 2D contour plots as a function of crystal size and X-ray wavelength with or without air and solvent around the crystal [Figs. 1 ▸(*a*)–1 ▸(*d*) and S1(*a*)–S1(*d*)].

## Appendix B Theoretical calculation of diffraction volume and absorption correction of spherical crystals

The mean intensity is related to diffraction volume and dose absorbed by a spherical crystal by (Holton & Frankel, 2010 ▸)

and

*I*_0_ = incident X-ray flux density.

*V*_xtal_ = diffraction volume or illuminated volume, defined by equation (5).

λ = X-ray wavelength.

μ = absorption coefficient (6.31 mm^−1^ for 2.7 Å and 11.5 mm^−1^ for 3.3 Å).

= X-ray path length in crystal, *i.e.* thickness along incident beam (T1) and diffracted beam directions (T2) combined at a given scattering angle of . Here,  stands for the rotation of the diffracted beam.

= mean transmittance term for spherical crystal at a given scattering angle of .

*r* = radius of the spherical crystal (µm).

*b* = X-ray beam size (µm).

We calculated the linear absorption coefficient (*i.e.* μ) of lysozyme using *RADDOSE*-3*D* (Zeldin *et al.*, 2013 ▸). It implied, μ (λ → 3.3 Å) = 1.15 × 10^−2^ µm^−1^ or 11.5 mm^−1^ and μ (λ → 2.7 Å) = 6.31×10^−3^ µm^−1^ or 6.31 mm^−1^. Based on equations (3) and (4) and analytical μ values, a numerical integration method was adopted to precisely account for absorption effect in a spherical crystal that had been shaped by the laser and absorption-corrected mean intensity at a given scattering angle was calculated.

As shown in Fig. S7, we modelled the crystal as a homogenous sphere of radius *r*. The center of the sphere (O) defines the origin of the coordinate system and the X-ray beam from the synchrotron is placed on the *Z* axis. The square X-ray beam with height and width equal to *b* and of top-hat profile was assumed to be centered with the center of the sphere.

Equation (5) refers to two competing terms – *V*_xtal_ and . Since the illuminated volume is the intersection of sphere (crystal) and cuboid (top-hat beam), as presented in Fig. S7, it is difficult to analytically calculate volume integrals from equations (6) and (7). Instead integrals were calculated numerically with *Mathematica* (Wolfram Research Inc.). Assuming that diffraction happened at point (*x*,*y*,*z*) inside the illuminated volume, we can calculate the optical path of the X-ray beam inside the crystal  as the sum of two terms – the path along the incident beam to the point where the X-ray beam intersects inside the crystal [T1 in Fig. S7(*b*)] and the path along the diffracted beam from the point of interaction to the point where it exits from the spherical crystal [T2 in Fig. S7(*b*)] (Becker & Coppens, 1974 ▸). The length of T2 depends upon the scattering angle (θ) of the diffracted beam. In addition, because of rotation of the spherical crystal, each reflection or diffracted vector will rotate 360°, which is accounted for in equation (4) as the ϕ term. In order to calculate the  term, we used a geometric formula for intersection between a line and a sphere. In practice, the volume integral over *V*_xtal_ is calculated numerically as a triple integral with (−*b*/2, *b*/2), (−*b*/2, *b*/2) and (−*r*, *r*) as limits for *x*, *y* and *z*, respectively, but with the integral content multiplied by an extra term that equals 1, if point (*x*,*y*,*z*) is inside *V*_xtal_ and 0 if point (*x*,*y*,*z*) is outside *V*_xtal_.

The theoretically calculated diffracted intensities and the corresponding ratios between different sizes of spherical crystals at different scattering angles are provided in Table S6 for wavelengths of 2.7 and 3.3 Å. The example *Mathematica* script for absorption-corrected intensity for 50 µm spherical crystal at 0^0^ scattering angle, *I*(0^0^), is also provided in the Supporting information.
