# Supplementary material for: Long-wavelength native-SAD phasing: opportunities and challenges
Source: IUCrJ. 2019 Apr 1;6(Pt 3):373–86. doi: 10.1107/S2052252519002756 (PMC6503925; doi:10.1107/S2052252519002756)
Supplement: Supplementary file 1 [file m-06-00373-sup1.pdf]

IUCrJ

Volume 6 (2019)

Supporting information for article:

Long-wavelength native-SAD phasing: opportunities and challenges

Shibom Basu, Vincent Olieric, Filip Leonarski, Naohiro Matsugaki, Yoshiaki Kawano, Tomizaki Takashi, Chia-Ying Huang, Yusuke Yamada, Laura Vera, Natacha Olieric, Jerome Basquin, Justyna A. Wojdyla, Kay Diederichs, Masaki Yamamoto and Meitian Wang

Table S1 Data processing statistics of 9 × 360° T_2R-TTL datasets collected at 1.9 Å.

d_{min}	Nrefl	Nunique	Npossible	Completeness	R_{meas}	<I/σ >	$CC_{1/2}$	$CC_{1/2}$anom
13.19	67390	1323	1352	97.90%	5.90%	93.89	100.00%	91%
9.33	157926	2470	2470	100.00%	4.60%	106.38	100.00%	85%
7.62	212206	3196	3196	100.00%	5.00%	92.99	100.00%	80%
6.60	253288	3754	3754	100.00%	6.40%	76.41	100.00%	78%
5.90	291131	4293	4293	100.00%	7.60%	64.65	100.00%	71%
5.39	322369	4738	4738	100.00%	7.90%	63.97	100.00%	64%
4.99	347950	5116	5116	100.00%	7.90%	66.56	100.00%	54%
4.66	373662	5543	5543	100.00%	7.70%	70.05	100.00%	42%
4.40	394151	5856	5856	100.00%	7.90%	69.19	100.00%	37%
4.17	418549	6215	6215	100.00%	9.10%	60.76	100.00%	34%
3.98	439305	6548	6548	100.00%	10.20%	54.68	99.90%	25%
3.81	458093	6851	6851	100.00%	11.70%	48.57	99.90%	21%
3.66	476653	7129	7129	100.00%	13.50%	42.36	99.90%	19%
3.53	470000	7369	7369	100.00%	16.10%	35.08	99.90%	13%
3.41	436082	7651	7651	100.00%	18.80%	27.87	99.80%	10%
3.30	465054	7974	7974	100.00%	24.70%	22.36	99.70%	10%
3.20	504794	8200	8200	100.00%	31.00%	19.04	99.60%	8%
3.11	526570	8408	8408	100.00%	39.90%	15.16	99.40%	8%
3.03	512335	8677	8677	100.00%	49.50%	11.92	99.00%	5%
2.95	481942	8938	8936	100.00%	60.60%	9.14	98.20%	0%
total	7609450	120249	120276	100.00%	11.70%	43.03	100.00%	28%

Table S2 Data processing statistics of 14 × 360° T_2R-TTL datasets collected at 2.7 Å.

d_{min}	Nrefl	Nunique	Npossible	Completeness	R_{meas}	<I/σ >	$CC_{1/2}$	$CC_{1/2}$anom
13.19	116993	1319	1347	97.90%	9.30%	99.39	99.90%	95%
9.33	256445	2461	2461	100.00%	6.70%	108.56	100.00%	95%
7.62	338294	3188	3188	100.00%	6.40%	94.42	100.00%	94%
6.60	398814	3747	3747	100.00%	7.80%	77.36	100.00%	93%
5.90	453642	4266	4266	100.00%	9.30%	65.07	100.00%	90%
5.39	500871	4720	4720	100.00%	9.50%	64.49	100.00%	87%
4.99	526405	5095	5095	100.00%	9.10%	66.48	100.00%	83%
4.66	500661	5525	5525	100.00%	8.40%	66.46	100.00%	75%
4.40	568332	5834	5834	100.00%	8.50%	67.99	100.00%	72%
4.17	545767	6186	6186	100.00%	10.10%	57.28	100.00%	65%
3.98	591193	6512	6512	100.00%	11.80%	51.52	99.90%	58%
3.81	643498	6846	6846	100.00%	14.40%	45.68	99.90%	51%
3.66	670828	7081	7081	100.00%	18.00%	38.37	99.90%	45%
3.53	698849	7389	7389	100.00%	24.60%	30.15	99.80%	36%
3.41	712989	7604	7604	100.00%	34.00%	22.68	99.70%	30%
3.30	738075	7906	7906	100.00%	50.30%	15.77	99.30%	31%
3.20	760936	8168	8168	100.00%	71.00%	10.96	98.70%	30%
3.11	787313	8457	8457	100.00%	98.60%	7.16	96.40%	25%
3.03	704169	8581	8582	100.00%	129.70%	4.32	91.50%	28%
2.95	625401	8835	8887	99.40%	176.30%	2.41	75.50%	18%
total	11139475	119720	119801	99.90%	14.60%	39.06	100.00%	57%

Table S3 Data processing statistics of 2.7 Å datasets from spherical lysozyme crystals

50 μm

d_{min}	Nrefl	Nunique	Npossible	Completeness	R_{meas}	<I/σ >	$CC_{1/2}$	$CC_{1/2}$anom
7.27	2080	302	313	96.50%	2.60%	78.74	99.90%	98%
5.14	3944	564	572	98.60%	2.60%	65.13	100.00%	95%
4.20	5309	733	742	98.80%	2.30%	71.47	100.00%	94%
3.64	5452	768	880	87.30%	2.40%	67.33	100.00%	86%
3.25	5231	858	1005	85.40%	2.80%	53.79	99.90%	83%
2.97	5591	857	1104	77.60%	3.20%	45.08	99.90%	80%
2.75	5691	851	1194	71.30%	3.60%	38.59	99.90%	77%
2.57	4609	788	1287	61.20%	4.50%	29.77	99.90%	76%
2.42	5030	790	1405	56.20%	4.90%	29.33	99.80%	77%
2.30	4732	759	1398	54.30%	5.50%	25.42	99.80%	63%
total	47669	7270	9900	73.40%	2.90%	47.89	100.00%	85%

100 μm

d_{min}	Nrefl	Nunique	Npossible	Completeness	R_{meas}	<I/σ >	$CC_{1/2}$	$CC_{1/2}$anom
7.27	2000	295	310	95.20%	2.50%	86.57	99.90%	99%
5.14	3951	568	575	98.80%	2.40%	74.28	100.00%	97%
4.20	5313	732	742	98.70%	2.10%	81.4	100.00%	95%
3.64	5427	764	876	87.20%	2.00%	77.8	100.00%	89%
3.25	5239	853	1009	84.50%	2.30%	63.69	100.00%	88%
2.97	5578	851	1096	77.60%	2.60%	54.62	100.00%	88%
2.75	5707	851	1199	71.00%	2.90%	47.55	99.90%	88%
2.57	4546	773	1275	60.60%	3.60%	37.51	99.90%	82%
2.42	5063	794	1413	56.20%	3.80%	37.31	99.90%	82%
2.30	4723	756	1371	55.10%	4.50%	32.67	99.80%	75%
total	47547	7237	9866	73.40%	2.50%	56.84	100.00%	90%

200 μm

d_{min}	Nrefl	Nunique	Npossible	Completeness	R_{meas}	<I/σ >	$CC_{1/2}$	$CC_{1/2}$anom
7.27	2154	294	309	95.10%	4.30%	41.92	99.70%	94%
5.14	3932	564	569	99.10%	4.50%	33.69	99.80%	89%
4.20	5213	734	743	98.80%	4.50%	36.65	99.90%	81%
3.64	5135	747	860	86.90%	5.00%	34.76	99.80%	65%
3.25	4981	847	1005	84.30%	5.50%	28.14	99.80%	49%

2.97	5391	846	1094	77.30%	6.20%	24.57	99.80%	60%
2.75	5571	837	1186	70.60%	7.00%	21.26	99.70%	53%
2.57	4589	782	1283	61.00%	7.80%	16.83	99.50%	51%
2.42	4918	785	1390	56.50%	8.20%	16.97	99.50%	50%
2.30	4608	750	1382	54.30%	9.30%	14.7	99.30%	39%
total	46492	7186	9821	73.20%	5.60%	25.62	99.80%	68%

Table S4 Data processing statistics of 3.3 Å datasets from spherical lysozyme crystals.

50 μm

d_{min}	Nrefl	Nunique	Npossible	Completeness	R_{meas}	<I/σ >	$CC_{1/2}$	$CC_{1/2}$anom
8.85	1105	168	177	94.90%	3.70%	59.48	99.90%	98%
6.26	2221	316	317	99.70%	3.90%	50.43	99.90%	98%
5.11	2922	395	409	96.60%	3.80%	47.69	99.90%	97%
4.43	3038	449	488	92.00%	3.30%	46.42	99.90%	95%
3.96	2940	468	553	84.60%	3.20%	47.59	99.90%	90%
3.61	3044	463	611	75.80%	3.20%	45.94	99.90%	89%
3.35	3061	458	641	71.50%	3.40%	42.87	99.90%	88%
3.13	2547	468	733	63.80%	3.60%	35.25	99.90%	87%
2.95	2710	442	760	58.20%	4.00%	31.64	99.90%	87%
2.80	2639	402	782	51.40%	4.60%	30.27	99.90%	83%
total	26227	4029	5471	73.60%	3.50%	42.55	99.90%	93%

100 μm

d_{min}	Nrefl	Nunique	Npossible	Completeness	R_{meas}	<I/σ >	$CC_{1/2}$	$CC_{1/2}$anom
8.85	1098	167	177	94.40%	3.80%	62.68	99.80%	98%
6.26	2220	316	317	99.70%	3.80%	53.95	99.90%	98%
5.11	2935	395	409	96.60%	3.50%	51.77	99.90%	97%
4.43	3029	446	486	91.80%	3.10%	50.37	100.00%	96%
3.96	2947	470	555	84.70%	3.00%	51.3	99.90%	91%
3.61	3057	464	611	75.90%	2.90%	50.03	99.90%	90%
3.35	3057	457	640	71.40%	3.10%	47.22	99.90%	91%
3.13	2545	467	734	63.60%	3.30%	39.08	99.90%	88%
2.95	2709	442	760	58.20%	3.60%	35.39	99.90%	88%
2.80	2627	401	782	51.30%	4.10%	34.13	99.90%	85%
total	26224	4025	5471	73.60%	3.30%	46.44	99.90%	94%

200 μm

d_{min}	Nrefl	Nunique	Npossible	Completeness	R_{meas}	<I/σ >	$CC_{1/2}$	$CC_{1/2}$anom
8.85	1179	166	175	94.90%	5.30%	37.45	99.70%	95%
6.26	2263	318	319	99.70%	5.50%	28.89	99.80%	95%
5.11	2917	392	406	96.60%	5.50%	26.58	99.80%	91%
4.43	3001	444	487	91.20%	5.70%	26.1	99.80%	86%
3.96	2892	464	549	84.50%	5.70%	27.28	99.80%	69%

3.61	3020	466	615	75.80%	6.10%	26.17	99.70%	62%
3.35	2906	448	631	71.00%	7.10%	23.61	99.70%	62%
3.13	2505	463	733	63.20%	7.00%	20.06	99.70%	61%
2.95	2670	434	759	57.20%	7.80%	17.79	99.60%	63%
2.80	2577	395	774	51.00%	8.60%	16.71	99.50%	59%
total	25930	3990	5448	73.20%	6.30%	24.17	99.70%	79%

Table S5 Data processing statistics of 2.7 Å and 3.3 Å datasets from one spherical lysozyme crystals with matched reflections.

2.7 Å

d_{min}	Nrefl	Nunique	Npossible	Completeness	R_{meas}	<I/σ >	$CC_{1/2}$	$CC_{1/2}$anom
8.85	1096	168	177	94.90%	2.50%	83.55	99.90%	98%
6.26	1883	306	319	95.90%	2.70%	63.22	99.90%	97%
5.11	2733	395	409	96.60%	2.70%	60.68	99.90%	95%
4.43	2884	449	492	91.30%	2.50%	60.84	100.00%	94%
3.96	2914	466	549	84.90%	2.30%	64.42	100.00%	85%
3.61	2642	449	620	72.40%	2.50%	56.98	100.00%	85%
3.35	2520	456	641	71.10%	2.90%	50.58	99.90%	81%
3.13	2412	456	743	61.40%	2.70%	44.21	100.00%	80%
2.95	2661	435	755	57.60%	3.20%	40.11	99.90%	81%
2.80	2636	406	794	51.10%	3.60%	37.33	99.90%	79%
total	24381	3986	5499	72.50%	2.70%	54.22	100.00%	90%

3.3 Å

d_{min}	Nrefl	Nunique	Npossible	Completeness	R_{meas}	<I/σ >	$CC_{1/2}$	$CC_{1/2}$anom
8.85	1092	168	177	94.90%	3.60%	60.12	99.90%	98%
6.26	1868	307	317	96.80%	3.90%	47.18	99.90%	97%
5.11	2739	395	409	96.60%	3.80%	46.53	99.90%	97%
4.43	2898	448	488	91.80%	3.30%	45.79	99.90%	96%
3.96	2931	468	553	84.60%	3.20%	48.14	99.90%	90%
3.61	2620	442	611	72.30%	3.10%	44.03	99.90%	89%
3.35	2550	458	641	71.50%	3.30%	39.29	99.90%	86%
3.13	2373	451	733	61.50%	3.60%	35.15	99.90%	87%
2.95	2700	442	760	58.20%	4.00%	31.8	99.90%	86%
2.80	2635	404	784	51.50%	4.60%	30.39	99.90%	83%
total	24406	3983	5473	72.80%	3.50%	41.59	99.90%	93%

Table S6 Absorption corrected intensities and their ratios for spheres of 25, 50, and 100 μm radii respectively as calculated based on Eq. 5 and 6**.**

r (μm)	25	50	100
I_0, b = 50 (μm)	π/4 = 0.7854	1	1
I_0, b = 40 (μm)	0.9716	1	1
V_{xtal} b=40(μm)	58119	151120	315693
λ (Å) = 2.7			
<A(30^0)>	0.781705	0.558215	0.296942
I(30^0)	44142	84355.2	93742.3
**Ratio[30^0]** *	**1.0**	**1.91**	**2.12**
**Expt Measured ratio**	**1.0**	**~2.0**	**~1.9**
λ (Å) = 3.3			
<A(30^0)>	0.639616	0.345691	0.109369
I(30^0)	36118.4	52239.4	34527.0
**Ratio[30^0]**	**1.0**	**1.45**	**0.95**
**Expt. Measured ratio**	**1**	**~1.5**	**~0.7**

* The ratios are very similar in the 2θ range of 0 to 30°.

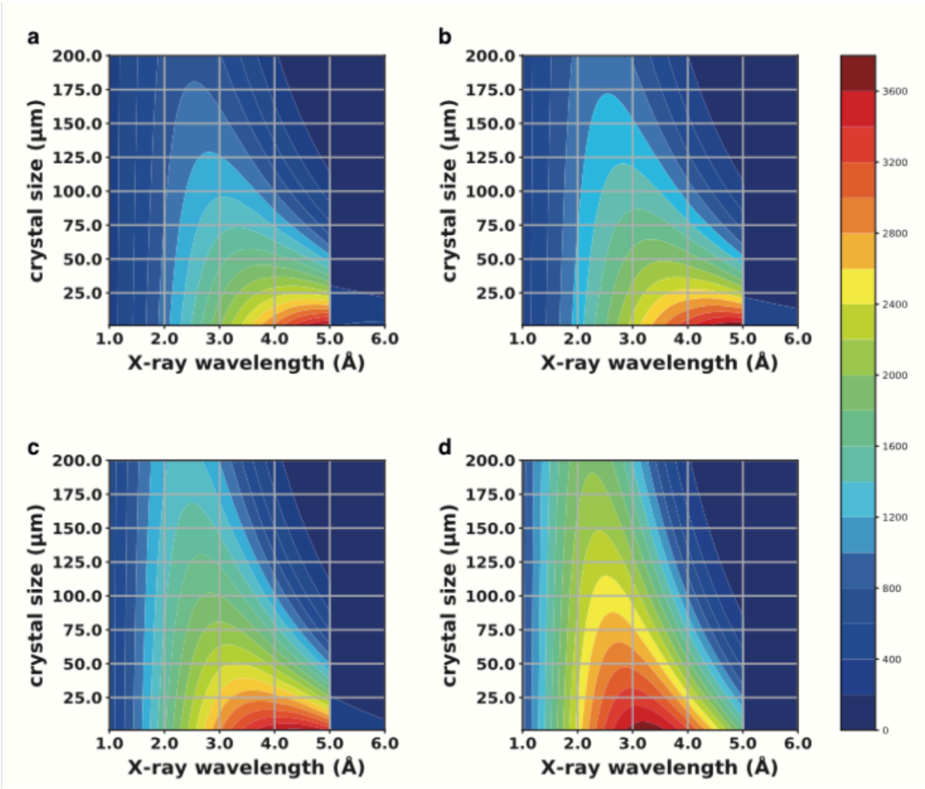

Figure S1 2-D contour plots of theoretical anomalous diffraction efficiency for sulfur atom as a function of X-ray wavelengths (X-axis) and crystal sizes (Y-axis). All plots assumed absence of air in the diffracted X-ray beam path. The X-ray absorption effect of various solvent thicknesses – **a.** 5 um, **b.** 10 um, **c.** 20 um, and **d.**50 um are displayed.

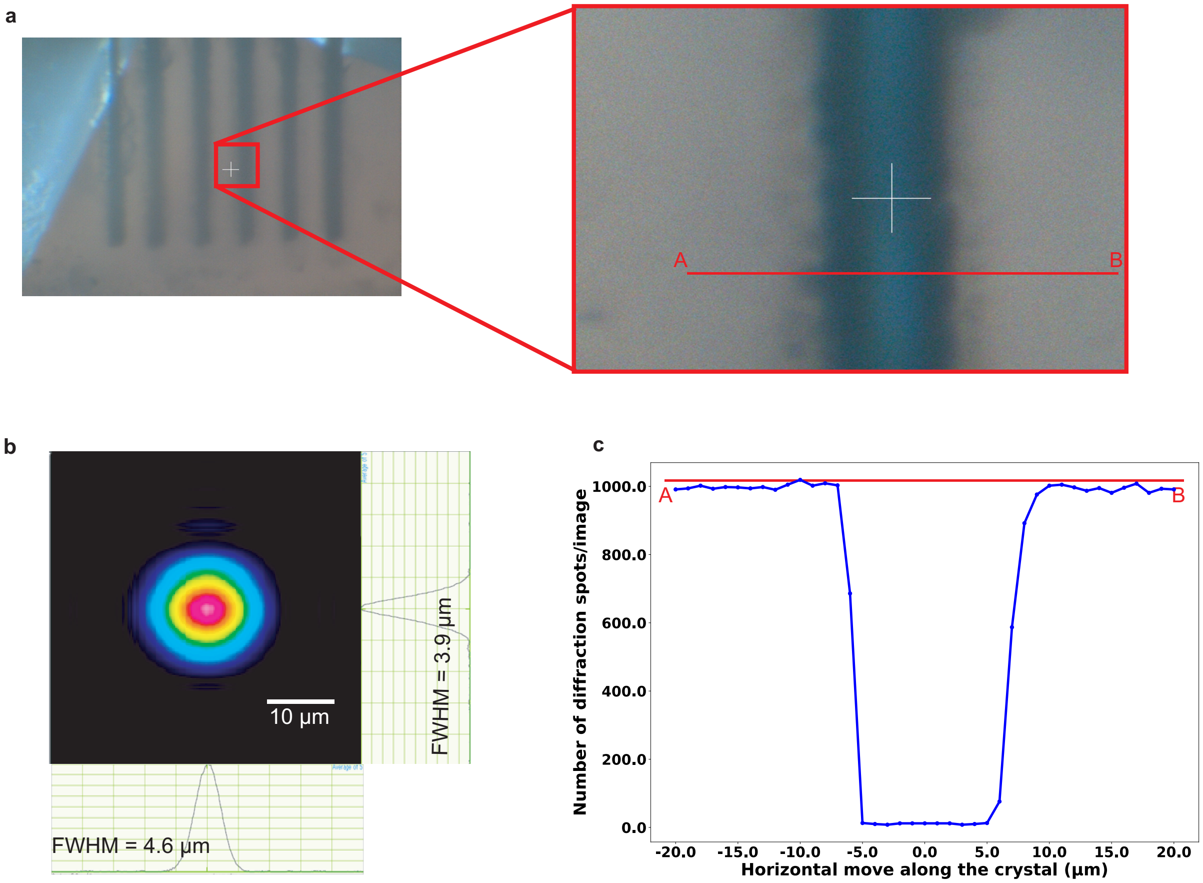

Figure S2 Evaluation of irradiated damage due to deep-UV laser-shaping on a cytochrome-c-oxidase crystal. a. On-axis view of the crystal, which is shaped into vertical lines using deep-UV laser system. The zoomed-in view shows how the line-shaped crystal was rastered from point A to B using a horizontal line (red). **b.** The 2D Gaussian beam profile for the deep-UV laser with a scale-bar (white). **c.** A plot, representing the decay of diffraction as the crystal is being rastered horizontally from A to B position (shown as red line) with Y-axis referring to the number of diffraction spots per image and X-axis referring to horizontal move of the crystal in µm.

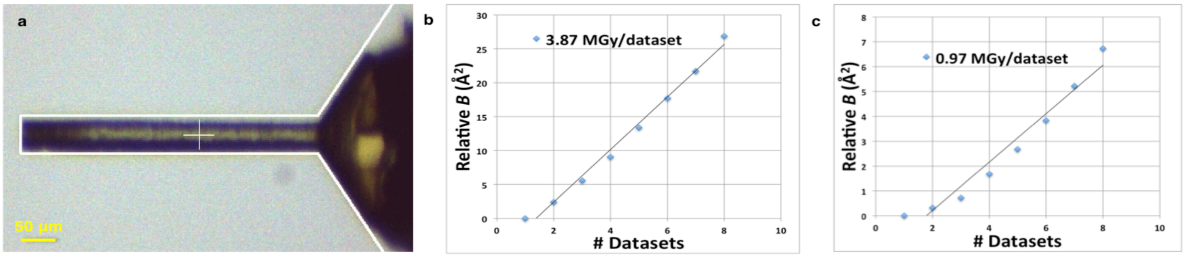

Figure S3 Measurement and estimation of dose normalization on cylindrically laser-shaped lysozyme crystal. a. Lysozyme crystal, shaped into 50 µm thick cylinder using a deep-UV laser. This crystal was used for dose estimation at 2.7 and 3.3 Å wavelengths, respectively. **b.** Relative *B* factors against no. of datasets at 2.7 Å wavelength with a linear curve-fitting (black line). **c.** Relative *B* factors against no. of datasets at 3.3 Å wavelength with a linear curve-fitting (black line).

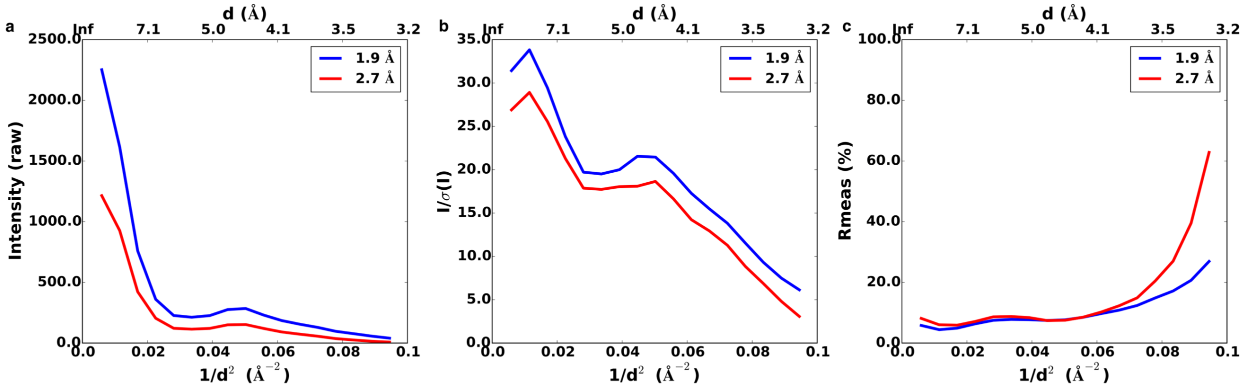

Figure S4 Comparison of statistics from a 360° dataset collected at 1.9 and 2.7 Å wavelength on a T_2R-TTL crystal. a. Observed diffracted intensities, **b**. I/σ(I) values, and **c.** R_{meas} values.

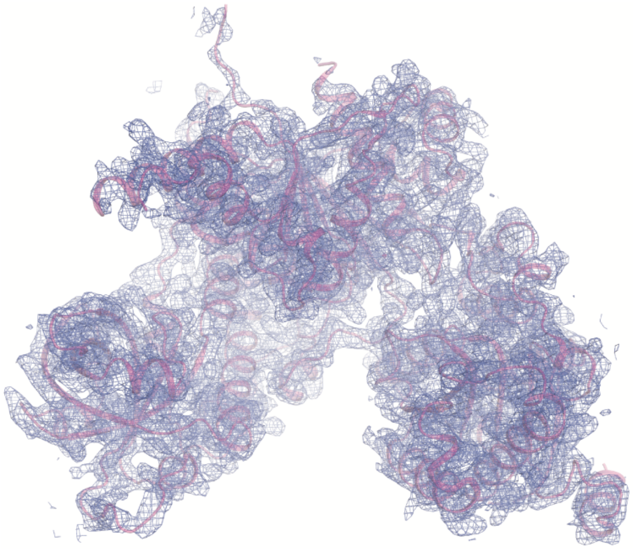

Figure S5 Experimental phasing map of Sen1. Experimental phasing map after density modification (shown in blue), contoured at 2.0 σ along with C-α trace.

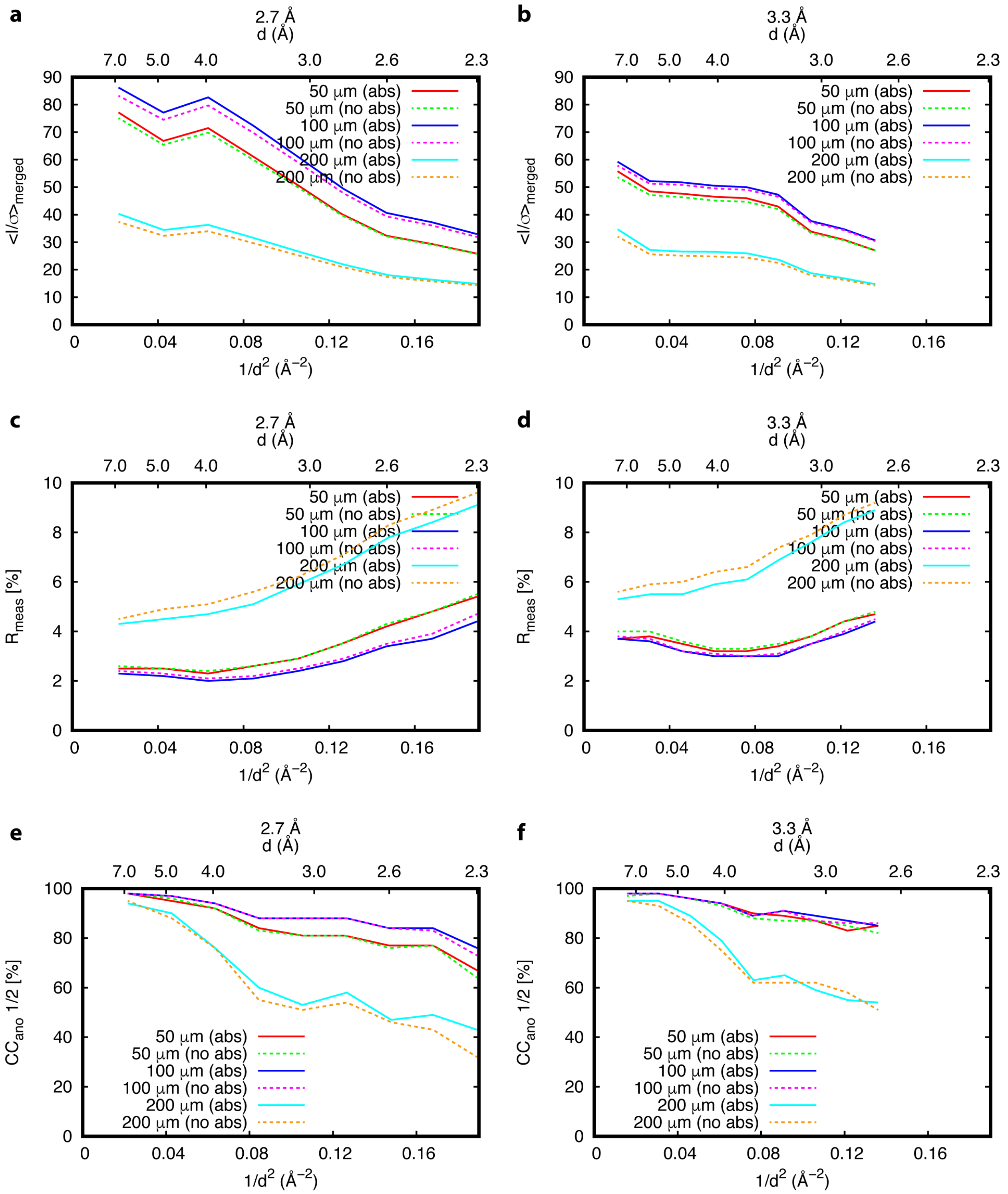

Figure S6 Data processing statistics with and without absorption correction from spherical lysozyme cystals.

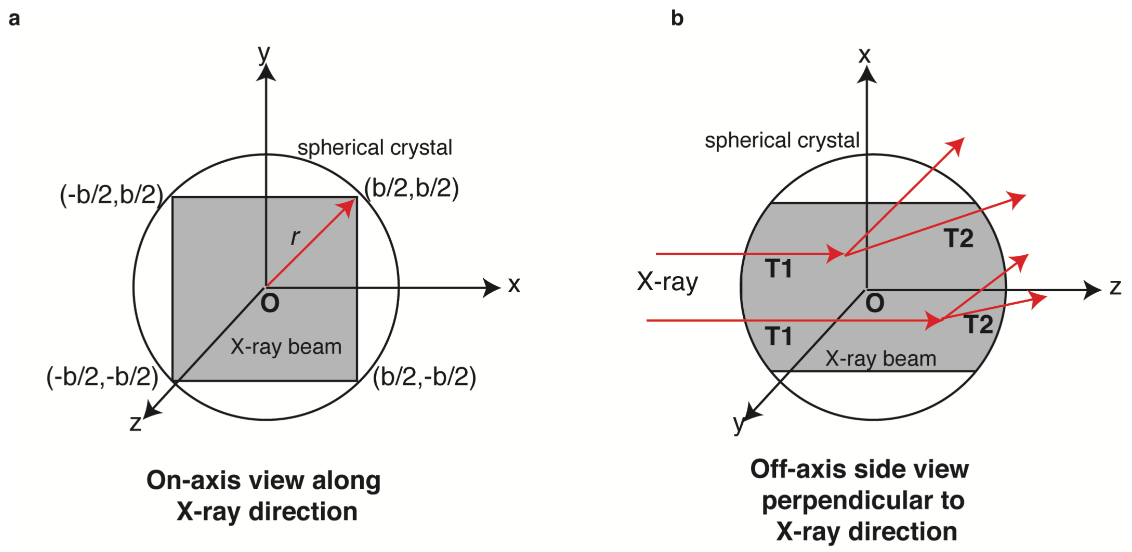

Figure S7 Geometrical representation of X-ray illuminated region with absorption paths on a spherical crystal. X-ray beam has a square projection (shown in gray) of length *b*. The origin of the coordinate system is at the center of the spherical crystal. The radius of spherical crystal is shown as *r*. **a.** On-axis view along the direction of X-ray beam. **b.** Off-axis side view, depicting the X-ray path through the crystal. T1 is the X-ray path length up to the point of interaction inside the crystal and T2 is the path length of the diffracted beam, which is from intersection point to exit point of the spherical crystal. T1 and T2 paths - both are shown in red colour.

Absorption correction script in Mathematica

```
r = 25; *radius of the spherical crystal*
b = 40; *X-ray beam size*
erx = 0.0; ery = 0.0; *erx and ery allow to simulate off center beam position*
I0 = 0.9716;
a = 0.32*10^-3;
xray = 3.3; *X-ray wavelength in Angstrom*
mu = a*(xray)^3; *linear absorption coefficient*

*Volume of a sphere calculation*
Volsphere = (4*Pi*r^3)/3;
*check if a point is inside or outside spherical crystal*
inSphere[x_, y_, z_] := If[x^2 + y^2 + z^2 > r^2, 0, 1]

*function to calculate intersection between a line and sphere*
intersection[o_, l_, dir_] := -Dot[o, l] + dir*Sqrt[Dot[o, l]^2 - Norm[o]^2 + r^2]

*see https://en.wikipedia.org/wiki/Line–sphere_intersection *
*T1 calculation: beam path inside the sphere up to the intersection point*
t1[x_, y_, z_] := -intersection[{x, y, z}, {0, 0, 1}, -1]
*T2 calculation: beam path from intersection point to the exit point of the sphere*
t2[x_, y_, z_, alpha_, phi_] := intersection[{x, y, z}, {Sin[alpha]*Cos[phi], Sin[alpha]*Sin[phi], Cos[alpha]}, 1]

*Total absorption path length = T1+T2*
path[x_, y_, z_, alpha_, phi_] := t1[x, y, z] + t2[x, y, z, alpha, phi]

Absorp[x_, y_, z_, alpha_, phi_] := Exp[-mu*path[x, y, z, alpha, phi]]

*Numerical integration to calculate illuminated volume due to X-ray and sphere interaction*
V = NIntegrate[inSphere[x, y, z], {z, -r, r}, {y, (-b/2.0) - ery, (b/2.0) - ery}, {x, (-b/2.0) + erx, (b/2.0) + erx}]

AbsInt[alpha_] := NIntegrate[inSphere[x, y, z]*Absorp[x, y, z, alpha, phi], {z, -r,r}, {y, (-b/2.0) - ery, (b/2.0) - ery},
{x, (-b/2.0) + erx, (b/2.0) + erx}, {phi, 0, Pi*2}]

PathAbsCorrInt[alpha_] := NIntegrate[inSphere[x, y, z]*path[x, y, z, alpha, phi]*
 Absorp[x, y, z, alpha, phi], {z, -r, r}, {y, (-b/2.0) - ery, (b/2.0) - ery}, {x, (-b/2.0) + erx, (b/2.0) + erx}, {phi, 0,
Pi*2}];

PathInt[alpha_] := NIntegrate[inSphere[x, y, z]*path[x, y, z, alpha, phi], {z, -r, r}, {y, (-b/2.0) + ery, (b/2.0) + ery},
{x, (-b/2.0) + erx, (b/2.0) + erx}, {phi, 0, Pi*2}];

T = PathAbsCorrInt[30*Pi/180.0]/AbsInt[30*Pi/180.0];

A = AbsInt[0*Pi/180]/(V*2*Pi);
Icorr = I0*V*A;
```

Supplementary Movie S1. Demonstration of the shaping procedure on a lysozyme crystal into different diameters of spheres using a deep-UV laser system.
